# Periodontal ligament tissues support neutrophil differentiation and maturation processes

**DOI:** 10.3389/fimmu.2024.1446541

**Published:** 2024-11-11

**Authors:** Guillermo Villagómez-Olea, Eileen Uribe-Querol, Francisco Javier Marichi-Rodríguez, Jorge Meléndez-Zajgla, Marco Antonio Alvaréz-Pérez, Carlos Rosales

**Affiliations:** ^1^ Laboratorio de Bioingeniería de Tejidos, División de Estudios de Posgrado e Investigación, Facultad de Odontología, Universidad Nacional Autónoma de México, Mexico City, Mexico; ^2^ Laboratorio de Biología del Desarrollo, División de Estudios de Posgrado e Investigación, Facultad de Odontología, Universidad Nacional Autónoma de México, Mexico City, Mexico; ^3^ Departamento de Ortodoncia, División de Estudios de Posgrado e Investigación, Facultad de Odontología, Universidad Nacional Autónoma de México, Mexico City, Mexico; ^4^ Laboratorio de Genómica Funcional, Instituto Nacional de Medicina Genómica, Mexico City, Mexico; ^5^ Departamento de Inmunología, Instituto de Investigaciones Biomédicas, Universidad Nacional Autónoma de México, Mexico City, Mexico

**Keywords:** neutrophil, periodontium, periodontal ligament, inflammation, granulopoiesis, single-cell RNA sequencing, periodontitis

## Abstract

**Introduction:**

Periodontal ligament is the soft connective tissue joining the roots of teeth with alveolar bone. The periodontal ligament presents significant cellular heterogeneity, including fibroblasts, endothelial cells, cementoblasts, osteoblasts, osteoclasts, and immune cells such as macrophages and neutrophils. These cells have crucial roles for periodontium homeostasis and function. However, certain cell types, such as neutrophils, remain poorly characterized in this tissue, despite their natural abundance and relevance in processes and diseases affecting the periodontal ligament.

**Methods:**

In order to characterize neutrophils present in periodontal ligament, and get some insight into their functions, single-cell RNA sequencing data from published reports was analyzed to integrate and create a comprehensive map of neutrophil heterogeneity within the murine periodontal ligament under steady-state conditions.

**Results:**

Four distinct neutrophil populations were identified based on their unique transcriptional signatures. Comparison and trajectory analysis revealed that these populations represent discrete stages of neutrophils undergoing maturation. These neutrophil populations were also classified, based on their granule content-associated signatures, as azurophil, specific, a transitional stage between specific and gelatinase (specific/gelatinase), and gelatinase. This reflects the sequential order of granule formation during neutrophil development (granulopoiesis) in the bone marrow.

**Discussion:**

Together, our findings indicate that the periodontal ligament may serve as a microenvironment where the ordered and sequential maturation of neutrophils takes place. This suggests that similarly to other niches, the murine periodontal ligament can support, to some extent, hematopoietic processes such as granulopoiesis.

## 1 Introduction

Periodontium, the main structure anchoring the tooth to the bone, is composed of diversity of mineralized and non-mineralized tissues such as the cementum, the alveolar bone, the gingiva, and the periodontal ligament (PDL) (1, 2). The plasticity of periodontal tissues is fashioned by the mesenchymal stem cell population in the PDL (3), which gives rise to three stromal lineages: periodontal fibroblasts, cementoblasts, and osteoblasts (4). In addition, PDL includes several other cell types, including epithelial cell rests of Malassez (5), neurovascular cells (6), and immune cells (7, 8). Among the innate immune cells found in the PDL, neutrophils actively maintain homeostasis by keeping bacteria in check (9, 10). Nevertheless, a number of periodontal pathogens can evade neutrophil microbicidal functions and promote periodontitis (11, 12).

Therefore, elucidating the nuances of neutrophil functions within the oral cavity is crucial for comprehending periodontal pathophysiology. However, characterizing neutrophils in the oral cavity has proven challenging due to their inherent complexity (13, 14) and the limited number of cells accessible for analytical techniques. The advances in single-cell technologies offer a powerful platform to examine the transcriptional landscape of many cells simultaneously. Particularly, single-cell RNA sequencing (scRNA-seq) has begun to unravel the diversity of neutrophil states in different tissues and pathological conditions (15–17). In order to characterize neutrophils in the oral cavity, their ontogeny, and alterations associated with diseases like periodontitis, three scRNA-seq datasets from published reports were integrated and reanalyzed to create a comprehensive map of neutrophil heterogeneity within the murine periodontal ligament under steady-state conditions. Four distinct neutrophil populations were identified based on their unique transcriptional signatures. These populations constitute discrete stages of neutrophils undergoing maturation that correspond to the sequential order of granule formation during neutrophil development in the bone marrow. These data suggest that the PDL serves as a microenvironment where the ordered and sequential differentiation of neutrophils takes place.

## 2 Methods

To identify the composition of the murine PDL, three datasets from independent scRNA-seq published studies on the murine PDL were selected for bioinformatic reanalysis. After combining and normalizing datasets, a total of 12,677 high-quality cells, with 20,033 mouse genes were detected across all cells. Differentially expressed genes and UMAP analysis resulted in a cell Atlas of the PDL. Various neutrophil subpopulations were identified and subjected to trajectory inference (pseudotime) and gene ontology (GO) analyses. Complete details of materials and methods can be found in **Supplementary Materials** and Methods.

## 3 Results

### 3.1 Single-cell RNA-seq integration reveals neutrophil heterogeneity in the periodontal ligament

**Figure 1:**
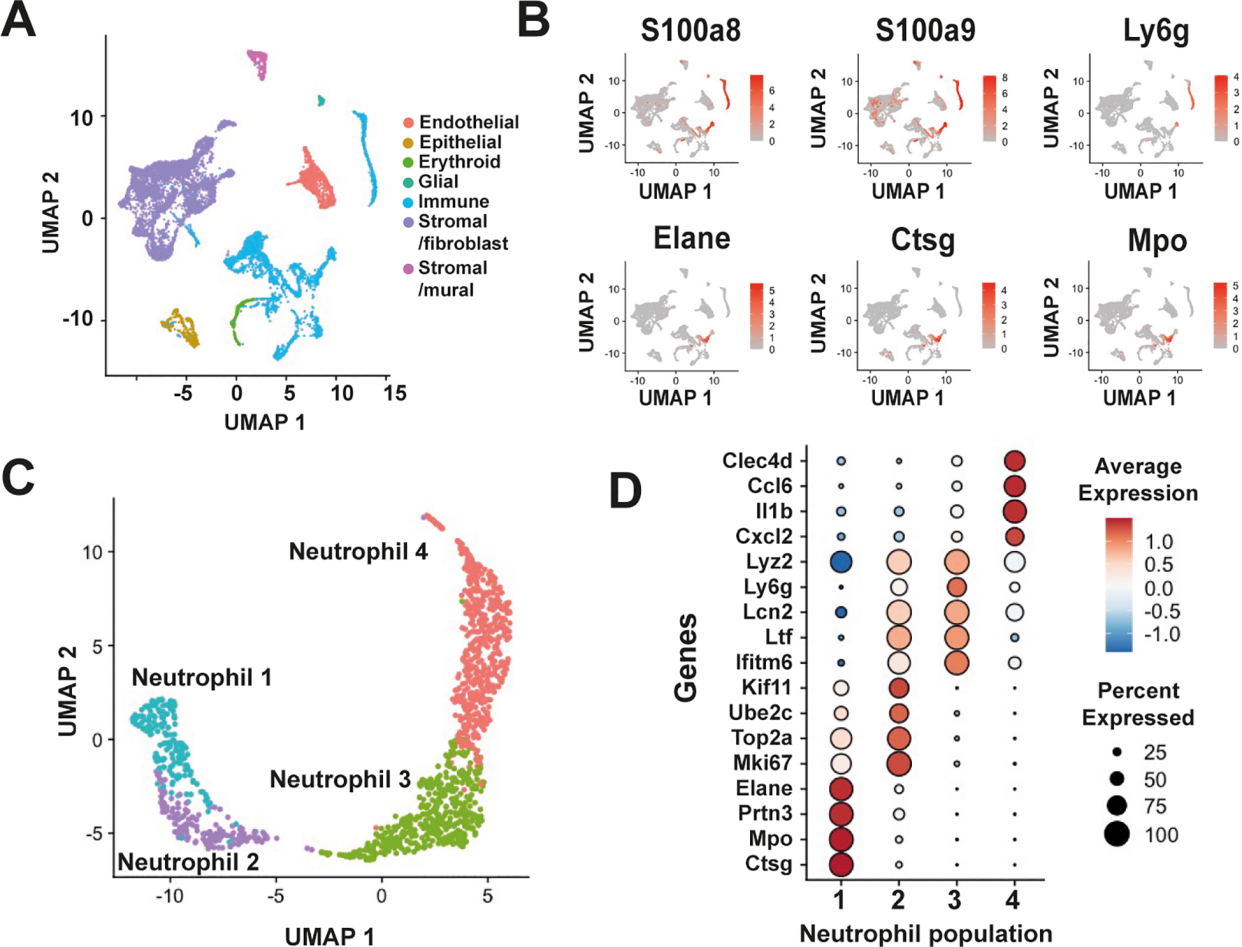
Single-cell RNA-seq integration reveals neutrophil heterogeneity in the murine periodontal ligament. **(A)** UMAP plot of the murine PDL atlas, showing the major cell populations colored by cell type: endothelial, epithelial, erythroid, glial, stromal/fibroblast, stromal/mural, and immune cells. **(B)** Neutrophils within the immune cell population were identified by the expression of neutrophil markers such as S100a8 and S100a9 (S100 calcium binding proteins A8 and A9). More restricted expression markers include Ly6g (lymphocyte antigen 6, family member G), Elane (neutrophil-expressed elastase), Ctsg (cathepsin G), and Mpo (myeloperoxidase). **(C)** Isolation and reanalysis partitioned neutrophils into four distinct subpopulations. **(D)** Dot plots showing the expression of genes associated with each neutrophil population in the PDL.

Three datasets from independent scRNA-seq studies of the murine PDL were integrated to identify the composition of neutrophils in this tissue (4, 18, 19) (**Supplementary Figure S1A**). These datasets correspond to healthy, young adult mice, in which the PDL was completely formed. To conduct the bioinformatics analysis, strict quality control filters were applied before using Canonical Correlation Analysis as the integration method, ensuring the removal of batch effects (**Supplementary Figure S1B**). Cell populations were projected by using UMAPs. This resulted in the establishment of an atlas of the murine PDL (n=12,677 cells) distributed across 43 clusters (**Supplementary Figure S1C**). Cell clusters corresponding to various cell types were identified by expression of pan-lineage markers (**Supplementary Figure S1D**), resulting in seven major cell types: endothelial, epithelial, erythroid, glial, immune, stromal/fibroblasts, and stromal/mural (**Figure 1A**). Immune cells, a well-defined major group of CD45^+^ (coded for by Ptprc) cells represented one of the most prominently cell types (**Figure 1A**). Among immune cells, neutrophils (1354 cells), were identified based on their expression of S100a8, S100a9, Ly6g, Elane, Ctsg, and Mpo (**Figure 1B**). None of the immune cells expressed genes such as Kit, Cd34, Ly6a, and Slamf1, associated to hematopoietic stem cells (HSC), nor the gene Sox4, associated to granulocyte monocyte progenitor (GMP) cells, indicating the absence of these cell types in the murine PDL (**Supplementary Figure S2A**).

Isolation and unsupervised clustering analysis with varying resolution parameters resulted in the grouping of neutrophils into three to nine clusters (**Supplementary Figure S2B**). The 4-cluster model, however, defined neutrophil subpopulations more clearly with minimal overlap. Consequently, we selected this model and designated the subpopulations as “Neutrophil 1,” “Neutrophil 2,” “Neutrophil 3,” and “Neutrophil 4” (**Figure 1C**). We also confirmed that each dataset contributed to these four neutrophil subpopulations (**Supplementary Figure S2C**). Cells in the “Neutrophil 1” cluster exhibited a transcriptional signature associated with early stages of neutrophil maturation (**Figure 1D**). This signature (Ctsg^+^, Mpo^+^, Ptrn3^+^, Elane^+^) reflects the primary/azurophil granule population and it is indicative of committed proliferative neutrophil precursors (preNeu) (20) or G1-neutrophils (21), as defined previously. The “Neutrophil 2” population displayed high expression of genes associated with cell proliferation, including Mki67, Top2a, Ube2c, and Kif11. Additionally, this population exhibited elevated expression of Ltf, Lcn2, Ifitm6, and Lyz2, genes associated with secondary granule proteins (22) (**Figure 1D**). The “Neutrophil 3” population was identified as a post-mitotic cluster where expression of genes associated with secondary or specific granules persisted (**Figure 1D**). Notably, an increase in the expression of Ly6g, a marker for identification of neutrophils (23), was observed in this cluster. However, expression of this marker was transient and mainly confined to Neutrophil 2 and Neutrophil 3 clusters (**Figure 1D**). In the “Neutrophil 4” population, an increase in the expression of markers associated with mature neutrophils (21, 22), such as Il1b, as well as the chemokines Ccl6, and Cxcl2 was observed (**Figure 1D**). Additionally, this population exhibited expression of Clec4d, a marker of activated neutrophils (24) (**Figure 1D**).

### 3.2 Neutrophil populations in the periodontal ligament display a continuum of maturation process

**Figure 2:**
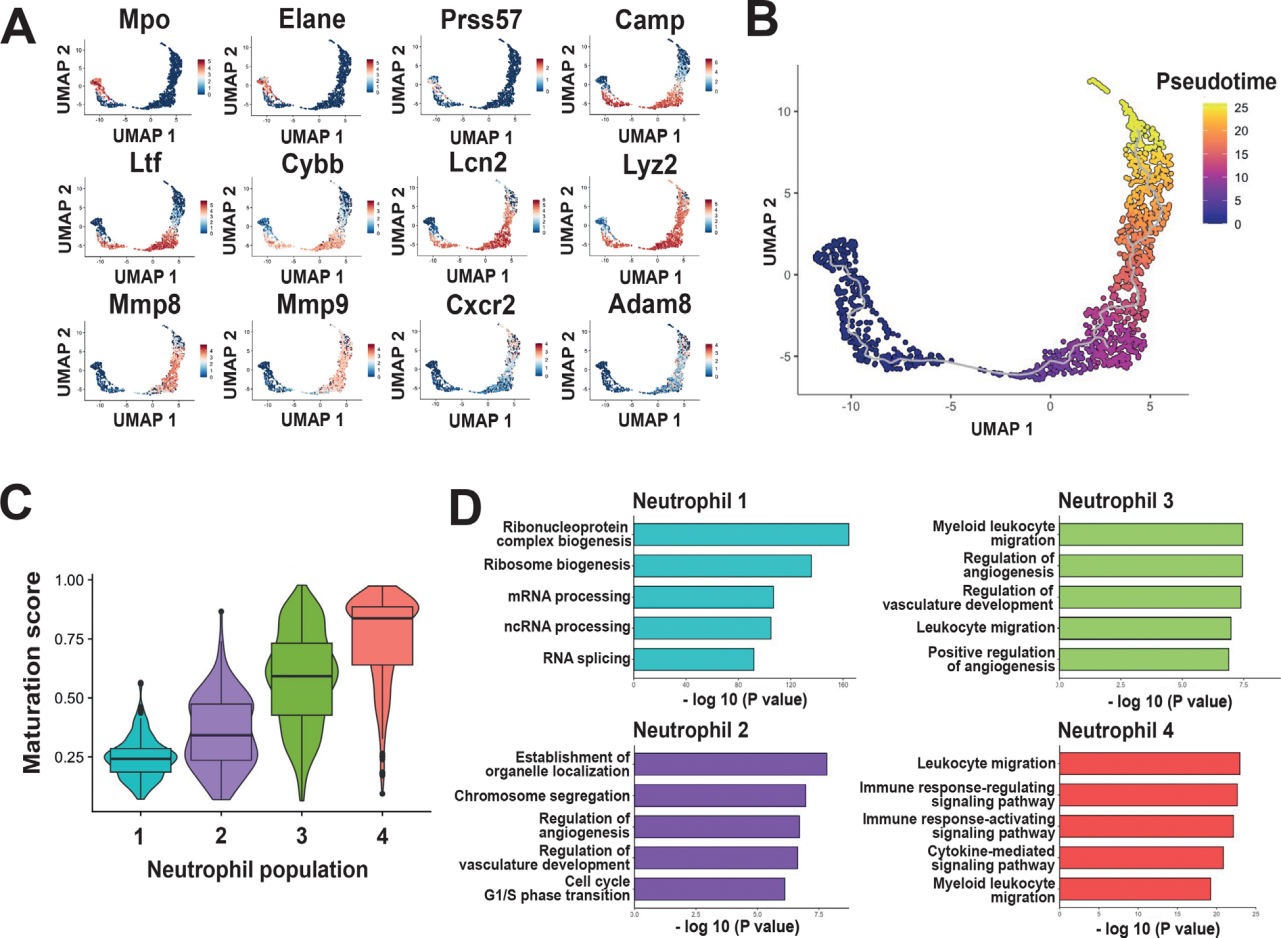
Neutrophil populations in the periodontal ligament (PDL) display a continuum of maturation process. **(A)** Expression of genes associated with neutrophil granules: Mpo, Elane, and Prss57 (genes linked to primary granules), Camp, Ltf, Cybb, Lcn2, and Lyz2 (genes linked to secondary granules), Mmp8, Mmp9, Cxcr2, and Adam8 (genes linked to tertiary granules and secretory vesicles). **(B)** Pseudotime analysis with Monocle3 identified a chronologically ordered differentiation trajectory of neutrophil populations in the PDL, with the Neutrophil 1 population representing the earliest stage of development and the Neutrophil 4 population representing states of greater maturation. **(C)** Maturation analysis indicated that the Neutrophil 1 population included cells with a low maturation score, while Neutrophil 2, 3, and 4 populations included cells with progressively higher maturation scores. **(D)** Gene Ontology (GO) analysis differentially expressed genes (DEGs) among the four neutrophil populations shows distinct transcriptional signatures associated with each neutrophil population.

Our results hinted that the neutrophil populations found in the PDL represented a continuum of cells undergoing a maturation process. To further explore this possibility, the expression of genes associated with neutrophil granular content was evaluated. Expression of Mpo and Elane (genes linked to primary granules) was associated to the Neutrophil 1 population (**Figure 2A**). A slight expression of Prss57, a gene linked to primary granules was also detected in the Neutrophil 1 population (**Figure 2A**). Expression of Camp, Ltf, and Cybb (genes linked to secondary granules) was found in both Neutrophil 2 and Neutrophil 3 populations, but it was higher in the Neutrophil 3 population (**Figure 2A**). In addition, expression of Lcn2 and Lyz2 (genes also considered to be markers of secondary granules) extended from the Neutrophil 2 population through the Neutrophil 4 population (**Figure 2A**). Similarly, expression of Mmp8 and Mmp9 (genes linked to tertiary granules) was found overlapping Neutrophil 3 and Neutrophil 4 populations (**Figure 2A**). Expression of these genes decreased at the distal end of the Neutrophil 4 population, particularly for Mmp8 and Mmp9 (**Figure 2A**). Conversely, at the distal end of the Neutrophil 4 population, there was an increase in expression of the gene Cxcr2, which codes for a chemokine receptor important for the maturation and effector function of neutrophils; and of the gene Adam8, which codes for a metalloproteinase associated with tertiary granules and secretory vesicles (**Figure 2A**). Together, these results suggested that the observed neutrophil populations correspond to cells sequentially maturing within the PDL.

To support the hypothesis that neutrophil populations in the PDL recapitulate maturation stages, trajectory analysis using Monocle3 was performed. This analysis identifies the interrelationships and developmental progress between cell populations by constructing a trajectory that reflects their main differentiation paths (25). Trajectory analysis revealed a continuous transition of neutrophil populations in the PDL, stretching from the Neutrophil 1 population at pseudotime 0 to the Neutrophil 4 population at pseudotime 25 (**Figure 2B**). This result showed that neutrophils in the PDL are ordered through a linear trajectory, without major branches. Moreover, maturation analysis indicated that the Neutrophil 1 population included cells with a low maturation score, while Neutrophil 2, 3, and 4 populations included cells with progressively higher maturation scores (**Figure 2C**).

In addition, to elucidate biological processes associated with each neutrophil population, Gene Ontology (GO) analysis was performed. The Neutrophil 1 population showed a signature associated with mechanisms of control and regulation of transcription and translation (**Figure 2D**). The Neutrophil 2 population showed a signature associated with cell cycle processes, suggesting that these cells are actively proliferating (**Figure 2D**). Also, Neutrophil 2 cells had a signature associated to regulation of angiogenesis (**Figure 2D**). The Neutrophil 3 population showed a signature associated with leukocyte migration, indicating that these are in fact more mature cells (**Figure 2D**). Neutrophil 3 cells also had a signature associated to vascular development and angiogenesis (**Figure 2D**). The Neutrophil 4 population showed a signature associated with leukocyte migration, cytokine signalling, and immune response (**Figure 2D**). All these processes are related to more mature non-proliferating neutrophils.

### 3.3 Neutrophils undergo transcriptional changes in the periodontal ligament during periodontal disease

**Figure 3:**
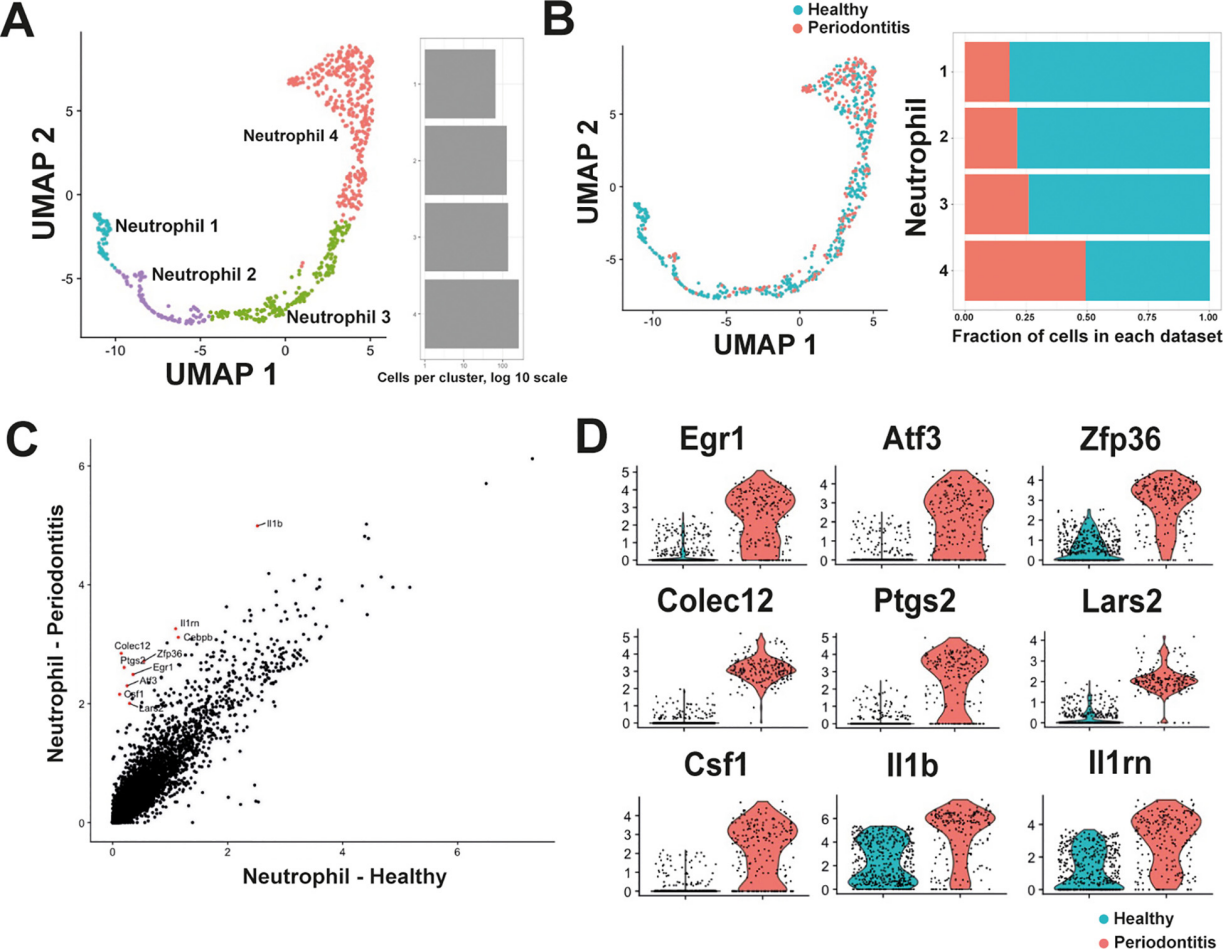
Neutrophils undergo transcriptional changes in the periodontal ligament (PDL) during periodontal disease. **(A)** Analysis and integration of the scRNA-seq dataset comprising cells from PDL in homeostasis and PDL with periodontitis (19), identified four neutrophil populations similar to those initially identified in the construction of the periodontal ligament atlas. **(B)** Quantification of cells comprising each neutrophil population revealed that Neutrophil 1 cluster consisted predominantly of cells from healthy PDL, while the Neutrophil 4 cluster contained almost similar proportions of cells from healthy and from inflamed PDL. The Neutrophil 4 population consisted mostly of mature neutrophils. **(C)** Average expression analysis identified differentially expressed genes between neutrophils from healthy PDL and neutrophils from PDL with periodontitis. In periodontitis, increased expression of Il1b (interleukin 1 beta), Il1rn (interleukin 1 receptor antagonist), Cebpb (CCAAT/enhancer binding protein beta), Colec12 (collectin sub-family member 12), Ptgs2 (prostaglandin-endoperoxide synthase 2; also known as cyclooxygenase 2), Zfp36 (zinc finger protein 36), Egr1 (early growth response 1), Atf3 (activating transcription factor 3), Csf1 (colony stimulating factor 1), and Lars2 (leucyl-tRNA synthetase, mitochondrial) was observed. **(D)** Violin plot analysis of the upregulated genes during periodontitis. The genes Egr1, Atf3, and Zfp36 code for transcription factors that regulate expression of genes associated with control of inflammation. Colec12, Ptgs2, and Lars2 code for proteins involved in activation of mature neutrophils. The genes Csf1, Il1b, Il1rn code for cytokines that regulate inflammation.

In healthy conditions, four populations of neutrophils could be clearly identified in the PDL (**Figure 1C**). However, there was no information on how these neutrophil populations could change during oral disease. Periodontitis is a serious disease characterized by infection and inflammation of the PDL (8), and neutrophils are important cells to maintain homeostasis in the PDL (9, 12). Therefore, in order to elucidate transcriptional alterations experienced by neutrophils in the PDL during disease, published data (19) comprising two datasets of PDL cells: one representing the PDL in homeostasis (n = 934 cells) – previously included in our initial analysis – and another representing the PDL under periodontitis (n = 1007 cells), were reanalyzed. The second dataset was generated from a murine teeth-ligation model that induces a robust acute inflammation in the periodontium (26). Isolated and integrated neutrophils from both conditions resulted in the same four neutrophil populations identified in our initial characterization (**Figure 3A**).

However, important changes in both cell proportion and gene expression were observed in neutrophils during periodontitis. The Neutrophil 1 population comprised mainly cells from healthy PDL, while the Neutrophil 4 population, included almost similar proportions of cells from healthy and from inflamed PDL (**Figure 3B**). The presence of cells within the four neutrophil populations during periodontitis suggested that the same differentiation trajectory is maintained under inflammatory conditions, albeit with an accelerated maturation process. This finding aligns with observations from bacterial infections, where neutrophils are primed for enhanced functionality without significantly altering overall heterogeneity (21). These changes in neutrophil maturation were also accompanied by changes in gene expression. Comparing the Average global expression profile of neutrophils from healthy PDL versus neutrophils from PDL with periodontitis revealed increased expression of genes such as Il1b, Il1rn, Cebpb, Colec12, Ptgs2, Zfp36, Egr1, Atf3, Csf1, and Lars2 in periodontitis (**Figure 3C**).

Among these upregulated genes during periodontitis (**Figure 3D**), Egr1, Atf3, and Zfp36 code for transcription factors that regulate expression of genes associated with control of inflammatory responses (27–29). Colec12, and Ptgs2 code for a scavenger receptor C-type lectin (30) and for cyclooxygenase (31), respectively. Lars2 codes for a mitochondrial leucyl-tRNA synthetase involved in mitochondrial metabolism (32). These proteins are all involved in activation of mature neutrophils during early immune responses. The genes Csf1, Il1b, Il1rn code for cytokines that regulate inflammation (33, 34) (**Figure 3D**). Together, these results suggest that during periodontitis, the maturation process of neutrophils is altered, leading to an increased presence of more mature cells and enhanced expression of genes associated with inflammation in these mature neutrophils.

## 4 Discussion

In this study, an atlas of cells within the murine periodontal ligament (PDL) was established., enabling the identification of four distinct neutrophil populations based on their unique transcriptional signatures. These populations represent discrete stages of neutrophil maturation, corresponding to the sequential order of granule formation during neutrophil development in the bone marrow.

To create an atlas of cells of the murine PDL, three independent single-cell RNA sequencing datasets obtained from adult murine PDL under steady-state conditions (4, 18, 19) were integrated. This approach resulted in the development of a comprehensive reference atlas of cells (n=12,677), which were distributed in 43 clusters. Among these cell clusters seven major cell types were identified, including endothelial, epithelial, erythroid, glial, immune, stromal/fibroblasts, and stromal/mural cells (**Figure 1A**). Immune cells represented the second largest major cell type in the PDL, underscoring their crucial role in maintaining health in the oral cavity (7, 35).

Neutrophils, are now recognized as the main type of immune cells in oral tissues (9, 10, 36, 37), and in perfect agreement, we found that neutrophils comprise about half of all immune cells. This reinforces the neutrophil status as the predominant innate immune cell in healthy periodontal tissues (12). The current model indicates that neutrophils migrate into the oral cavity as mature cells from the circulating blood (9, 12, 36). However, our result suggested that there are neutrophils in early maturation stages in the PDL. Further exploration showed that neutrophils from the PDL could indeed be partitioned into four distinct populations (**Figure 1C**). This implied that mature neutrophils migrating into the periodontal tissues could exist in four distinct phenotypes with possible different functions (13), most likely induced by cues from the periodontal tissue environment (38). This idea, however, was not supported by further analysis of gene expression. Instead, neutrophil populations sequentially mirrored proliferative states transitioning into mature, non-proliferative neutrophils (**Figure 1D**). The less mature neutrophil population (Neutrophil 1) exhibited a transcriptional signature consistent with the previously described unipotent progenitor neutrophil G1 cells (21), and also with the committed proliferative neutrophil precursor (preNeu) cells (20). Also, the Neutrophil 1 population had expression of genes associated with formation of primary (azurophilic) neutrophil granules (39, 40). Neutrophil 2 population expressed genes associated with cell cycle processes, suggesting that these cells are actively proliferating. Neutrophil 2 cells also had a transcriptional signature (Lcn2, Ifitm6, Ltf, and Lyz2 genes) associated with secondary (specific) granules. Thus, our results provide the first evidence of immature, proliferating neutrophils present in the PDL.

Neutrophil 3 and Neutrophil 4 populations were characterized as non-proliferating cells, representing more mature phases of neutrophils. Specifically, Neutrophil 3 was identified as an intermediate, postmitotic population expressing genes associated with both secondary and tertiary granules, while Neutrophil 4 was characterized as mature neutrophils. Expression of Mmp8 and Mmp9 (genes linked to tertiary granules) was found overlapping Neutrophil 3 and Neutrophil 4 populations, showing again the greater maturation stage of these cells (21). Neutrophil 4 cells also exhibited upregulation of genes associated with mature neutrophils, including Il1b, Ccl6, and Cxcl2, similarly to previous reports of mature neutrophils (21, 22). Additionally, these cells expressed Clec4d, a marker of activated neutrophils (24) and associated with resolution of inflammation (41), a desired response in the periodontium, which is constantly exposed to pro-inflammatory stimuli. Interestingly, expression of Ly6g was found to be transiently expressed and downregulated in mature neutrophils (**Figure 1D**). This finding seems contradictory with the fact that Ly6G is considered a classical marker of mature neutrophils. However, it is known that expression of many Ly6 proteins often correlates with stages of differentiation (23), and newly generated, circulating neutrophils have lower Ly6G membrane expression (42). Therefore, the lower expression of Ly6g mRNA in the Neutrophi 4 population may reflect that the newly generated mature neutrophils have less Ly6G protein on their membrane (42). Another, explanation for the reduced expression of Ly6g mRNA in the Neutrophi 4 population is that similarly to some granule proteins (39, 43), Ly6G is produced during the Neutrophil 3 stage and the protein remains on the cell surface, despite lower mRNA levels. Hence, it is likely that neutrophils isolated from the PDL may already express some of the proteins associated with more mature cells, and then neutrophils may re-initiate synthesis in the PDL (16, 44). Together, these results show that neutrophils within the periodontal ligament represent different states of maturation. This idea was further corroborated by a trajectory analysis (25), which revealed the ordered differentiation of these neutrophil populations in the PDL, stretching from the Neutrophil 1 population with unipotent progenitor capacity, through the Neutrophil 2 population with proliferative capacity, to finally the Neutrophil 3 and Neutrophil 4 populations with non-proliferating, mature neutrophils. Therefore, neutrophils in the murine PDL correspond to neutrophils in the process of maturation and not to mature neutrophils that undergo a second burst of transcriptional activity when migrating into tissues as previously suggested (43). Together these data suggest that the murine PDL can support extramedullary hematopoietic processes (45, 46). In addition to the liver, other organs such as spleen, lungs, and lymph nodes can support hematopoiesis (47), particularly in various pathological conditions such as some bacterial infections (48, 49) and cancer (50). Now we have found that the murine PDL can also support extramedullary hematopoiesis in healthy conditions. This finding is in agreement with previous reports indicating that the PDL is a stem cell niche, where stem cells contribute to the renewal of periodontal tissues (6, 51). Because the PDL is a tissue under constant physical and bacterial stress, it makes sense that this tissue can also support the proliferation, and maturation of neutrophils, which are essential for oral health (9, 12).

The origin of neutrophils in the PDL has not been identified, but it most likely is the alveolar bone. Previous studies have indicated that circulating neutrophils correspond to cells in more advanced stages of maturation, with almost no presence of precursor neutrophils (21, 22). This would imply that neutrophil precursors in the PDL are not recruited from circulating blood. Thus, it is more likely that these precursor cells derive from the alveolar bone marrow, which is very close to the PDL. It is also possible that alveolar bone marrow cannot efficiently support a complete differentiation process and in consequence, neutrophil precursors are liberated. A comprehensive comparison of neutrophil heterogeneity in PDL, alveolar bone marrow, and long bone marrow will help defining the origin of neutrophils in the PDL.

Although, our study clearly established four distinct neutrophil populations in the PDL, other reports have identified at least 7 subgroups of neutrophils (21), while others suggest that neutrophils exist in transitional states, forming a continuum rather than discrete groups of cells (22). Whether discrete cell populations or a continuum of cells exist is an open question and consequently this remains an active area of research. Both ideas are not mutually exclusive, thus it is possible that while neutrophils transit from a cell stage (discrete population) to the next, cells in various transitional steps could be detected. Our data suggest that in the murine PDL, neutrophils follow a continuous maturation program with clear separate cell stages. This provides a solid foundation for understanding neutrophil heterogeneity in oral tissues. However, we acknowledge the limitations of scRNA-seq techniques that preclude us from capturing all possible cell types or stages. scRNA-seq is unable to reliably detect low-abundance transcripts. Thus, activated, aged, or apoptotic neutrophils having low transcriptional activity would be underrepresented in data sets. Also, phenotype changes due to post-transcriptional modifications of molecules, will also not be detected. In addition, not all RNA molecules in a cell are necessarily translated into proteins (52, 53).

In this study, we also compared neutrophils in the PDL from healthy mice and from mice with periodontitis. In both conditions, the number of neutrophils was about the same (**Figure 3**). This was somewhat surprising, since in periodontitis a greater number of neutrophils is expected in periodontal tissues (12, 54). Our inability to detect more neutrophils in periodontitis may be attributed to the challenges associated with sequencing mature neutrophils, as they have lower transcript content and are more difficult to isolate and manipulate (55, 56). Despite this, our analysis was able to reveal significant changes in the subsets of neutrophils present in the PDL during periodontitis. Although, the four populations of neutrophils were conserved both in healthy and inflamed PDL, an important reduction in the proportion of cells corresponding to the early developmental stages of neutrophils was detected in periodontitis (**Figure 3**). This suggests that in response to infection, there is an acceleration of maturation processes, leading to a reduction of neutrophil immature populations to generate more mature cells.

In conclusion, in this study an atlas of cells within the murine periodontal ligament was established. Among immune cells, neutrophils represent the predominant cell type. Four distinct neutrophil populations were identified based on their unique transcriptional signatures. These populations constitute discrete stages of neutrophils undergoing a maturation process that correspond to the sequential order of granule formation during neutrophil maturation in the bone marrow. Also, during periodontitis the same trajectory of neutrophil differentiation persists, but there is also an important increase in the number of more mature neutrophils.

## Data Availability

Publicly available datasets were analyzed in this study. This data can be found here: Count matrices from Gene Expression Omnibus (GEO) under accession numbers GSE197828, GSE168450, and GSE160358.
