# Supplementary material for: Periodontal ligament tissues support neutrophil differentiation and maturation processes
Source: Front Immunol. 2024 Nov 11;15:1446541. doi: 10.3389/fimmu.2024.1446541 (PMC11586715; doi:10.3389/fimmu.2024.1446541)
Supplement: Supplementary file 1 [file DataSheet1.pdf]

Periodontal ligament tissues support neutrophil differentiation and maturation processes

Guillermo Villagómez-Olea[1], Eileen Uribe-Querol[2], Francisco Javier Marichi-Rodríguez[3], Jorge Meléndez-Zajgla[4], Marco Antonio Alvaréz-Pérez[1], Carlos Rosales[5*]

[1] Laboratorio de Bioingeniería de Tejidos, División de Estudios de Posgrado e Investigación, Facultad de Odontología, Universidad Nacional Autónoma de México, Mexico City, 04510, Mexico

[2] Laboratorio de Biología del Desarrollo, División de Estudios de Posgrado e Investigación, Facultad de Odontología, Universidad Nacional Autónoma de México, Mexico City, 04510, Mexico

[3] Departamento de Ortodoncia, División de Estudios de Posgrado e Investigación, Facultad de Odontología, Universidad Nacional Autónoma de México, Mexico City, 04510, Mexico

[4] Laboratorio de Genómica Funcional, Instituto Nacional de Medicina Genómica, Mexico City, 14080, Mexico

[5] Departamento de Inmunología, Instituto de Investigaciones Biomédicas, Universidad Nacional Autónoma de México, Mexico City, 04510, Mexico

2 MATERIALS AND METHODS

2.1 Periodontal Ligament (PDL) single-cell RNA-sequencing (scRNA-seq) data sets

To identify the composition of the murine PDL, three datasets from independent scRNA-seq published studies on the murine PDL were selected for bioinformatic reanalysis [1-3] (Supplementary Figure S1A). Count matrices from Gene Expression Omnibus (GEO) under accession numbers GSE197828, GSE168450, and GSE160358 were downloaded. Only matrices corresponding to healthy, young adult mice, where the PDL was completely formed were used. Matrices from PDL disease or development were excluded, unless indicated otherwise. Count matrices were loaded and analyzed using Seurat (v5) [4] in R program (v4.3.2) and Rstudio (v2023.12.0 + 369) as the Integrated Development Environment.

2.2 Quality control and processing of scRNA-seq datasets

Upon loading each dataset, Seurat objects were created. Low-quality cells were removed through quality control analysis, filtering, and selection processes based on criteria such as sequencing depth and mitochondrial content. Due to variations in data quality, each dataset was filtered using sample-specific cutoffs, a strategy recommended by good practice guides [5] and employed by other researchers [6]. Subsequently, the datasets were combined and normalized using the NormalizeData function with default parameters to account for differences in sequencing depth

across cells. We obtained a total of 12,677 high-quality cells, with 20,033 mouse genes detected across all cells.

2.3 Data integration and visualization

The datasets were integrated using the Seurat Integration Pipeline. The steps were performed using the functions implemented in the Seurat package with default parameters, including NormalizeData, FindVariableFeatures, ScaleData, and RunPCA. Subsequently, we identified nearest neighbors on the first 30 principal component analysis (PCA) dimensions and performed clustering at a resolution of 1.5. To visualize the data before batch correction, we applied Uniform Manifold Approximation and Projection (UMAP) [7] on the same 30 dimensions. For dataset integration, we used Canonical Correlation Analysis (CCA) via the IntegrateLayers function. Post-integration, the layers were re-joined using JoinLayers function on the "RNA" assay. To compare and determine the correct integration, we used the same parameters both to identify the nearest neighbors and to perform clustering. This approach allowed us to verify the correct integration and removal of batch effects (Supplementary Figure S1B).

2.4 Identification of DEGs (Differentially Expressed Genes) in the PDL atlas

For identifying cluster-specific markers in the Periodontal Ligament Atlas, we used the FindAllMarkers function to identify differentially expressed genes (DEGs) in all clusters. This function employs the Wilcoxon rank-sum test by default for statistical testing. The parameters were set to include only positive markers, with a minimum percentage threshold of 0.25 and a log-fold change threshold of 0.25. To determine the most significant markers, we grouped the markers by cluster and selected the top markers based on the average log2 fold change.

2.5 Cluster cells identification and labelling

Cell clusters corresponding to various cell types were identified by expression of the following genes. For stromal cells, Cola1 (collagen, type I, alpha 1) and Pdgfra (platelet derived growth factor receptor alpha); for mural cells, Rgs5 (regulator of G-protein signaling 5) and Myh11 (myosin, heavy polypeptide 11, smooth muscle); for epithelial cells, Krt5 (keratin 5) and Krt14 (keratin 14); for endothelial cells, Pecam1 (platelet/endothelial cell adhesion molecule 1) and Cdh5 (cadherin 5); for glial cells, Sox10 (SRY (sex determining region Y)-box 10) and Plp1 (proteolipid protein (myelin) 1); for pro-erythroblast cells, Gypa (glycophorin A); and for immune cells, Ptprc (protein tyrosine phosphatase receptor type C) (Supplementary Figure S1D). Among immune cells, neutrophils were identified by using unbiased automated cell type annotation with SingleR [8], using as reference the Immunological Genome Project (ImmGen) [9], and MouseRNAseq [10] databases; and by the expression of murine neutrophil markers including S100a8 (S100 calcium binding protein A8), S100a9 (S100 calcium binding protein A9), Ly6g (lymphocyte antigen 6, family member G), Elane (neutrophil-expressed elastase), Ctsg (cathepsin G), Cd81 (tetraspanin-28), Itgam (integrin alpha M), Mmp8 (matrix metallopeptidase 8), Mmp9 (matrix metallopeptidase 9), and Cd33 (Siglec-3). Expression of specific neutrophil granule proteins including Mpo (myeloperoxidase), Camp (cathelicidin antimicrobial peptide), Ltf (lactotransferrin), Prtn3 (proteinase 3), Prss57 (serine protease 57), and Cybb (cytochrome b-245, beta polypeptide; also known as gp91phox), was also examined. Likewise, expression of genes associated with mature neutrophils, such as Cxcr2 (C-X-C motif chemokine receptor 2), and Adam8 (a disintegrin and

metallopeptidase domain 8) was examined. In addition, expression of genes identifying haematopoietc stem cells (HSC), such as Kit (KIT proto-oncogene receptor tyrosine kinase; also known as CD117), Cd34 (CD34 antigen), Ly6a (lymphocyte antigen 6 family member A; also known as Sca-1), and Slamf1 (signaling lymphocytic activation molecule family member 1; also known as CD150), as well as expression of genes identifying granulocyte monocyte progenitor (GMP) cells, such as Sox4, (SRY (sex determining region Y)-box 4), was assessed.

2.6 Isolation and reanalysis of neutrophil subclusters from scRNA-seq PDL atlas

After identifying specific neutrophil clusters based on previously mentioned markers combined with automatic cell labeling approaches, these clusters were isolated for further detailed analysis. Principal component analysis (PCA) was performed on the isolated neutrophil clusters. The optimal number of principal components (PCs) was determined using an elbow plot (ElbowPlot), selecting the number of PCs based on the cumulative variance explained and the elbow point where the percentage of variance explained by successive PCs dropped significantly (https://hbctraining.github.io/scRNA-seq/lessons/elbow_plot_metric.html). Nearest neighbors were identified using the FindNeighbors function with the integrated canonical correlation analysis (CCA) reduction and the selected PCs. Clustering of neutrophil subpopulations was performed at different resolutions (0.1, 0.2, 0.3, 0.4, 0.5, 1.0), with a resolution of 0.2 ultimately chosen as optimal (Supplementary Figure S2B). To visualize the neutrophil subpopulations, UMAP was run using the integrated CCA reduction on the selected PCs. Dimensional plots were generated to display the clustering results, showing the distinct neutrophil subpopulations and allowing for further exploration of their transcriptional profiles. To identify conserved markers for each neutrophil population, the FindConservedMarkers function was used. For each neutrophil cluster, we set 'ident.1' to the specific neutrophil population to analyze, and 'ident.2' to NULL for an all-group comparison. The RNA assay and the data slot were used for normalized expression values, with a minimum of 3 cells per group required for the analysis. This function performs statistical tests including the Wilcoxon rank sum test for individual comparisons and combines p-values across groups using Fisher's method. This method identifies robust markers consistently expressed across the 3 datasets employed in our analysis, allowing for enhanced characterization of neutrophil subpopulations.

2.7 Developmental trajectory inference

Data from identified neutrophil populations were transferred from Seurat to Monocle 3 object format. Dimensional reduction and trajectory inference were then performed using Monocle3 (v3.0) to plot the hypothetical differentiation lineage trajectory [11, 12]. Initially, the Seurat object containing neutrophil clusters was converted to a CellDataSet object. Cell metadata, gene metadata, and count data were extracted using colData, fData, and counts functions, respectively. Gene names were assigned to the gene metadata for clarity. We then incorporated clustering information and UMAP coordinates from Seurat into the Monocle3 object. Partitions were assigned uniformly to all cells, and cluster identities were transferred based on the active identities from the Seurat object. UMAP embeddings from Seurat were also included. Trajectory inference was then carried out using the learn graph function without partition constraints. Cells were ordered in pseudotime using the order cells function, specifying the UMAP reduction method and selecting root cells from a specific neutrophil cluster.

2.8 Gene Ontology (GO) analysis

To obtain insights into the biological processes associated with the identified neutrophil populations, we performed gene ontology (GO) analyses [13]. Differentially expressed genes (DEGs) obtained using the FindAllMarkers function in Seurat were used as input for the analysis. Parameters were defined to detect significantly upregulated genes in each cluster. GO analysis was performed using the clusterProfiler package [14]. Specifically, we used the enrichGO function to identify overrepresented GO terms associated with DEGs. The analysis allowed us to characterize the biological functions and processes significantly enriched in each neutrophil subpopulation.

2.9 Maturation score analysis

To evaluate the maturation status of neutrophil populations, we computed a score based on a neutrophil maturation signature as previously described [6]. This maturation signature includes the genes: Retnlg, Ccl6, S100a6, Clec4d, Prr13, Cebpb, Slpi, S100a11, Btg1, Cxcr2, and Fth1. We used the UCell package [15], which employs a gene signature scoring method based on the Mann-Whitney U statistic. The UCell method calculates gene signature scores by first ranking gene expression values for each cell and then applying the U statistic to determine the enrichment of the gene set.

2.10 Differential expression analysis between healthy PDL and periodontitis

To identify differentially expressed genes (DEGs) between healthy PDL and periodontitis-affected PDL, we reanalyzed the Zhao et al. [3] datasets (GSE160358, GSM5115470) corresponding to samples from healthy PDL and PDL affected with periodontitis. After integrating the datasets and defining cell clusters corresponding to neutrophils in the integrated healthy and periodontitis profiles, we used the AverageExpression function to obtain the average expression of both healthy and disease neutrophil cells. The results were visualized in Scatter and Violin plots, highlighting the genes that exhibit significant upregulation under the inflammatory state.

2.11 References

1. Iwayama T, Sakashita H, Takedachi M, Murakami S. Periodontal tissue stem cells and mesenchymal stem cells in the periodontal ligament. *Jpn. Dent. Sci. Rev.* (2022) 58: 172-8. doi: 10.1016/j.jdsr.2022.04.001

2. Nagata M, Chu AKY, Ono N, Welch JD, Ono W. Single-cell transcriptomic analysis reveals developmental relationships and specific markers of mouse periodontium cellular subsets. *Front. Dent. Med.* (2021) 2: 679937. doi: 10.3389/fdmed.2021.679937

3. Zhao J, Birjandi AA, Ahmed M, Redhead Y, Olea JV, Sharpe P. Telocytes regulate macrophages in periodontal disease. *Elife* (2022) 11: e72128. doi: 10.7554/eLife.72128

4. Hao Y, Stuart T, Kowalski MH, Choudhary S, Hoffman P, Hartman A, et al. Dictionary learning for integrative, multimodal and scalable single-cell analysis. *Nat. Biotechnol.* (2024) 42: 293-304. doi: 10.1038/s41587-023-01767-y

5. Luecken MD, Theis FJ. Current best practices in single-cell RNA-seq analysis: a tutorial. *Mol. Syst. Biol.* (2019) 15: e8746. doi: 10.15252/msb.20188746

6. Xie X, Shi Q, Wu P, Zhang X, Kambara H, Su J, et al. Single-cell transcriptome profiling reveals neutrophil heterogeneity in homeostasis and infection. *Nat. Immunol.* (2020) 21: 1119-33. doi: 10.1038/s41590-020-0736-z

7. McInnes L, Healy J, Saul N, Gro.berger L, Melville J. UMAP: uniform manifold approximation and projection. *J. Open Sour. Soft.* (2018) 3: 861. doi: 10.21105/joss.00861

8. Aran D, Looney AP, Liu L, Wu E, Fong V, Hsu A, et al. Reference-based analysis of lung single-cell sequencing reveals a transitional profibrotic macrophage. *Nat. Immunol.* (2019) 20: 163-72. doi: 10.1038/s41590-018-0276-y

9. Heng TS, Painter MW, Immunological Genome Project Consortium. The Immunological Genome Project: networks of gene expression in immune cells. *Nat. Immunol.* (2008) 9: 1091-4. doi: 10.1038/ni1008-1091

10. Benayoun BA, Pollina EA, Singh PP, Mahmoudi S, Harel I, Casey KM, et al. Remodeling of epigenome and transcriptome landscapes with aging in mice reveals widespread induction of inflammatory responses. *Genome Res.* (2019) 29: 697-709. doi: 10.1101/gr.240093.118

11. Qiu X, Mao Q, Tang Y, Wang L, Chawla R, Pliner HA, et al. Reversed graph embedding resolves complex single-cell trajectories. *Nat. Methods* (2017) 14: 979-82. doi: 10.1038/nmeth.4402

12. Trapnell C, Cacchiarelli D, Grimsby J, Pokharel P, Li S, Morse M, et al. The dynamics and regulators of cell fate decisions are revealed by pseudotemporal ordering of single cells. *Nat. Biotechnol.* (2014) 32: 381-6. doi: 10.1038/nbt.2859

13. The Gene Ontology Consortium. The Gene Ontology Resource: 20 years and still GOing strong. *Nucleic Acids Res.* (2019) 47: D330-D8. doi: 10.1093/nar/gky1055

14. Wu T, Hu E, Xu S, Chen M, Guo P, Dai Z, et al. clusterProfiler 4.0: A universal enrichment tool for interpreting omics data. *Innovation (Camb.)* (2021) 2: 100141. doi: 10.1016/j.xinn.2021.100141

15. Andreatta M, Carmona SJ. UCell: Robust and scalable single-cell gene signature scoring. *Comput. Struct. Biotechnol. J.* (2021) 19: 3796-8. doi: 10.1016/j.csbj.2021.06.043

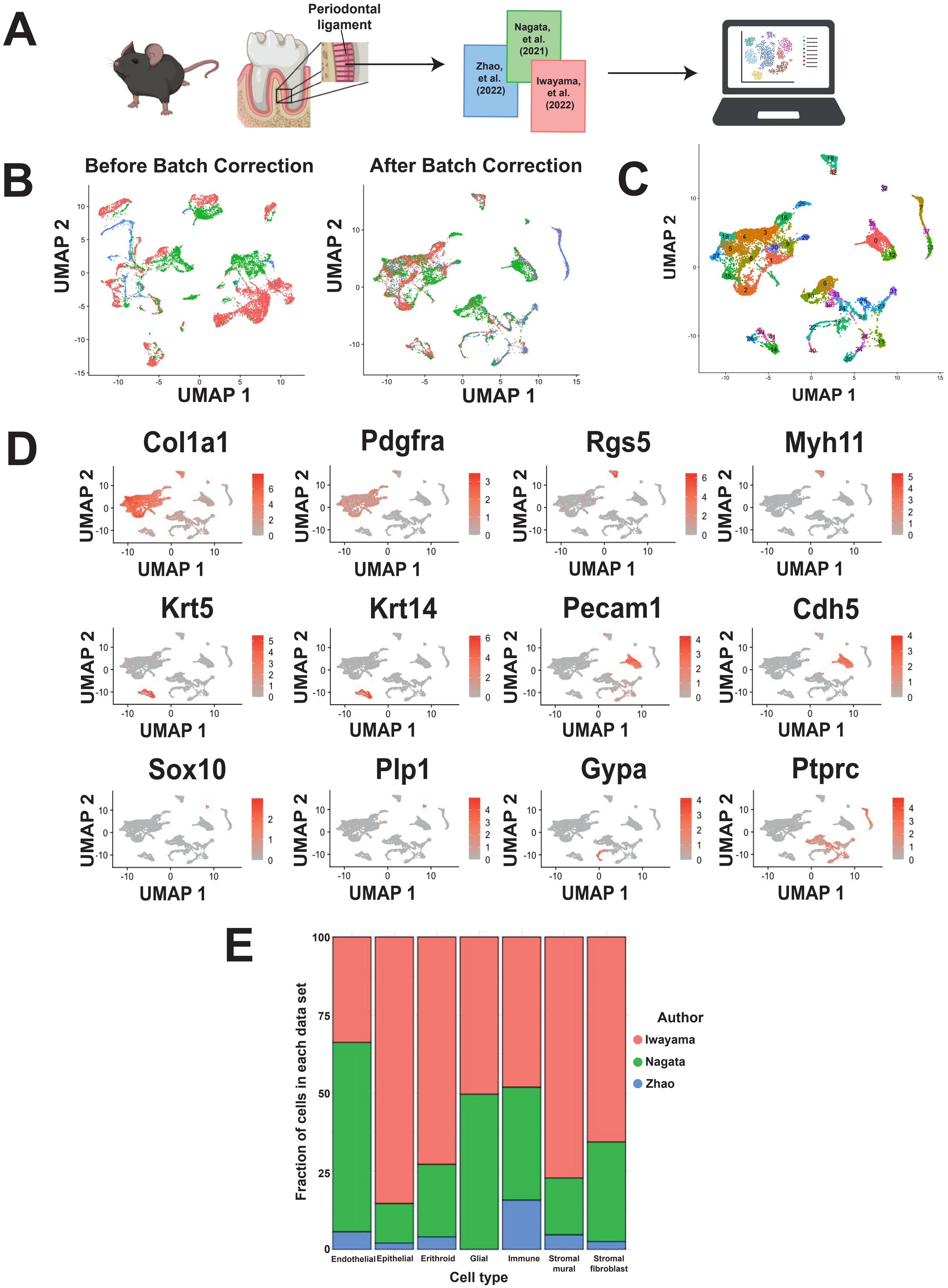
A
Periodontal ligament
Nagata, et al. (2021)
Zhao, et al. (2022)
Iwayama, et al. (2022)
B
Before Batch Correction
After Batch Correction
UMAP 2
UMAP 1
C
UMAP 2
UMAP 1
D
Col1a1
Pdgfra
Rgs5
Myh11
Krt5
Krt14
Pecam1
Cdh5
Sox10
Plp1
Gypa
Ptprc
UMAP 2
UMAP 1
E
Fraction of cells in each data set
Author
Iwayama
Nagata
Zhao
Endothelial
Epithelial
Erithroid
Glial
Immune
Stromal mural
Stromal fibroblast
Cell type

Supplementary Figure 1. *Identification of the different cell populations within the murine periodontal ligament.* **A)** To identify the composition of cells in the murine periodontal ligament (PDL) and to create an atlas of the murine PDL, three datasets from independent scRNA-seq studies (identified by author name) of the murine PDL were integrated [1-3]. These datasets correspond to healthy, young adult mice, in which the PDL was completely formed. **B)** UMAP (Uniform Manifold Approximation and Projections) visualization of the integrated PDL datasets before and after correction of batch effects. **C)** Atlas of the murine PDL. Cells were distributed across 43 clusters. **D)** Cell clusters corresponding to various cell types were identified by expression of the following genes: for stromal cells, Cola1 (collagen, type I, alpha 1) and Pdgfra (platelet derived growth factor receptor alpha); for mural cells, Rgs5 (regulator of G-protein signaling 5) and Myh11 (myosin, heavy polypeptide 11, smooth muscle); for epithelial cells, Krt5 (keratin 5) and Krt14 (keratin 14); for endothelial cells, Pecam1 (platelet/endothelial cell adhesion molecule 1) and Cdh5 (cadherin 5); for glial cells, Sox10 (SRY (sex determining region Y)-box 10) and Plp1 (proteolipid protein (myelin) 1); for pro-erythroblast cells, Gypa (glycophorin A); and for immune cells, Ptprc (protein tyrosine phosphatase receptor type C). **E)** Stacked bar plot showing the contribution of each dataset to the different cell types identified in the murine PDL atlas. Panel A was created with BioRender.com

1. Iwayama T, Sakashita H, Takedachi M, Murakami S. Periodontal tissue stem cells and mesenchymal stem cells in the periodontal ligament. *Jpn. Dent. Sci. Rev.* (2022) 58: 172-8. doi: 10.1016/j.jdsr.2022.04.001
2. Nagata M, Chu AKY, Ono N, Welch JD, Ono W. Single-cell transcriptomic analysis reveals developmental relationships and specific markers of mouse periodontium cellular subsets. *Front. Dent. Med.* (2021) 2: 679937. doi: 10.3389/fdmed.2021.679937
3. Zhao J, Birjandi AA, Ahmed M, Redhead Y, Olea JV, Sharpe P. Telocytes regulate macrophages in periodontal disease. *Elife* (2022) 11: e72128. doi: 10.7554/eLife.72128

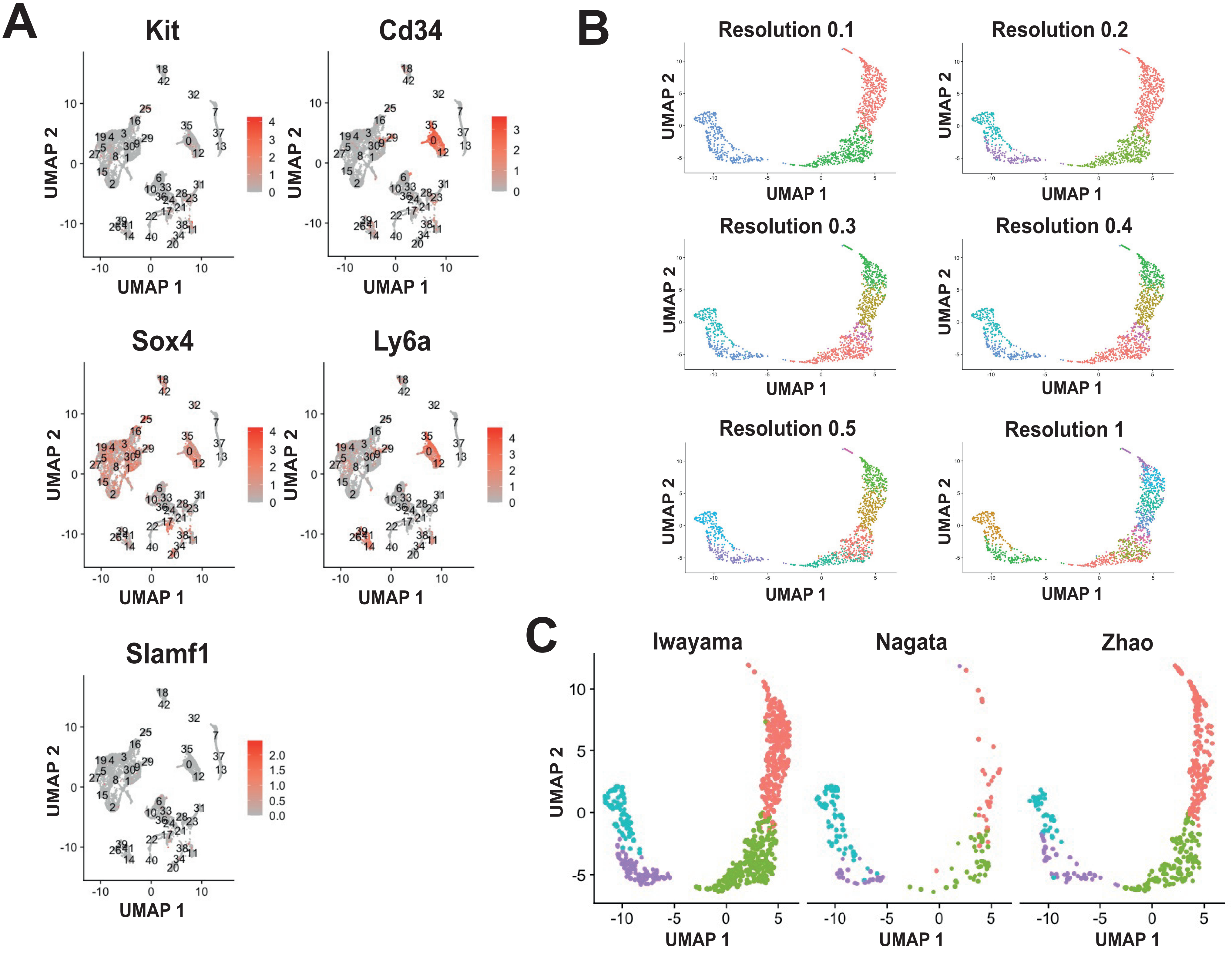
A
Kit
Cd34
Sox4
Ly6a
Slamf1
UMAP 1
UMAP 2
B
Resolution 0.1
Resolution 0.2
Resolution 0.3
Resolution 0.4
Resolution 0.5
Resolution 1
C
Iwayama
Nagata
Zhao

Supplementary Figure 2. Neutrophil populations within the murine PDL. **A)** None of the immune cells expressed genes such as Kit, Cd34, Ly6a, and Slamf1, associated to hematopoietic stem cells (HSC), nor the gene Sox4, associated to granulocyte monocyte progenitor (GMP) cells, indicating the absence of these cell types in the murine PDL. **B)** Isolation and unsupervised clustering analysis with different resolution parameters grouped neutrophils into three to nine clusters. Further analysis indicated that the 4-cluster model could partition neutrophils into four distinct populations. **C)** These four neutrophil populations could also be detected in each of the data sets included in the generation of the PDL atlas.
